# Effects of Chronic Moderate Alcohol Intake on Metabolic Phenotypes and Gut Microbiota in Lean and Obese Mice with Distinct Dietary Structures

**DOI:** 10.3390/nu17233658

**Published:** 2025-11-23

**Authors:** Jiu-Jiao Gao, Zi-Die Nian, Ning Li, Tong Wang, Han Sun, Mei Tang, Jian-Rui Li, Biao Dong, Jing-Chen Xu, Yue Gong, Xin-Yue Liu, Jian-Dong Jiang, Hu Li, Zong-Gen Peng

**Affiliations:** 1 CAMS Key Laboratory of Antiviral Drug Research, Institute of Medicinal Biotechnology, Chinese Academy of Medical Sciences & Peking Union Medical College, Beijing 100050, China; j2024080004@pumc.edu.cn (J.-J.G.); s2023010018@pumc.edu.cn (Z.-D.N.); s2024010018@student.pumc.edu.cn (N.L.); b2024010032@student.pumc.edu.cn (T.W.); b2023010026@pumc.edu.cn (H.S.); tangmei@imb.pumc.edu.cn (M.T.); lijianrui@imb.pumc.edu.cn (J.-R.L.); dongbiao@imb.cams.cn (B.D.); xujingchen@imb.pumc.edu.cn (J.-C.X.); gongyue@imb.pumc.edu.cn (Y.G.); s2023010043@pumc.edu.cn (X.-Y.L.); jiangjiandong@imb.cams.cn (J.-D.J.); 2 Key Laboratory of Biotechnology of Antibiotics, The National Health and Family Planning Commission (NHFPC), Institute of Medicinal Biotechnology, Chinese Academy of Medical Sciences & Peking Union Medical College, Beijing 100050, China; 3 State Key Laboratory of Bioactive Substance and Function of Natural Medicines, Institute of Medicinal Biotechnology, Chinese Academy of Medical Sciences & Peking Union Medical College, Beijing 100050, China

**Keywords:** metabolic and alcohol-associated liver disease, ethanol, dietary structure, metabolic phenotype, gut microbiota, risk assessment

## Abstract

Background: The 2023 Delphi consensus defined metabolic and alcohol-associated liver disease (MetALD), distinguishing between alcohol abuse and moderate consumption. Although alcohol abuse is known to accelerate fatty liver disease progression, the health effects of chronic moderate alcohol intake under different dietary conditions remain unclear. This study aimed to evaluate the impact of moderate alcohol consumption on metabolic phenotypes and gut microbiota/metabolites in lean and obese mice and to propose a model approximating MetALD features. Methods: C57BL/6J mice were fed either a low-fat diet (LFD) or a high-fat diet (HFD) for 12 weeks, with access to 10% (*v*/*v*) alcohol in drinking water. Systemic metabolic parameters, liver histopathology, inflammatory and fibrotic markers, gut microbiota composition, and the fecal metabolome were assessed. Results: In LFD-fed mice, 10% alcohol intake induced multiple metabolic alterations, including elevated serum triglycerides, reduced fasting blood glucose, and changes in hepatic lipid metabolism along with steatosis and inflammation—though further studies are required to confirm causality. When combined with HFD, alcohol did not significantly exacerbate most glucose/lipid metabolic disorders but markedly increased hepatic inflammatory cell infiltration and fibrosis progression. Alcohol consistently increased gut microbial α-diversity in both dietary groups, while downregulating beneficial metabolites such as amino acids (e.g., glutamine, histidine), their derivatives, and short-chain fatty acids. Correlation analyses associated these microbial and metabolic changes with altered amino acid/cholesterol metabolism and inflammatory/fibrotic phenotypes, particularly under HFD conditions. Conclusions: These findings suggest that chronic moderate alcohol intake presents distinct risks in lean and obese individuals with different dietary structures.

## 1. Introduction

Steatotic liver disease (SLD) is a broad term that primarily encompasses metabolic dysfunction-associated steatotic liver disease (MASLD) and alcohol-associated liver disease (ALD) [1,2], representing the leading cause of chronic liver disease worldwide [3,4]. The recent 2023 Delphi consensus established the term MASLD to replace nonalcoholic fatty liver disease (NAFLD) [5]. Concurrently, metabolic and alcohol-associated liver disease (MetALD) was also proposed to define individuals with metabolic dysfunction who intake moderate amounts of alcohol (20–50 g/day for females and 30–60 g/day for males) [5,6]. In contrast, alcohol-associated liver disease (ALD) is characterized by heavier alcohol intake (>50 g/day for females; >60 g/day for males) [5]. This refined classification distinguished liver injury primarily driven by heavy alcohol consumption (ALD) from that involving a combination of metabolic dysfunction and moderate alcohol intake. However, the beneficial or harmful impact of moderate alcohol consumption on the progression of metabolic diseases remains controversial [7,8,9,10,11], although animal studies conducted under specific experimental conditions and most clinical studies focusing on alcoholic populations suggest that it may significantly accelerate disease progression [12,13,14].

Existing rodent models for MetALD typically employ ad libitum drinking water with alcohol, along with weekly alcohol binges, or high alcohol concentrations (e.g., 20% *v*/*v* in water) administered via gavage [15,16], which exceeded the alcohol dose relevant to MetALD and potentially caused stress or mortality [9,12]. To specifically model the chronic and moderate alcohol exposure characteristic of MetALD, we developed a murine model utilizing ad libitum 10% (*v*/*v*) alcohol in drinking water without gavage, alongside a low or high-fat diet (LFD or HFD) for 12 weeks. Generally, a 30–60 g/day alcohol intake in male MetALD patients approximately corresponds to 6–12 g/kg/day in mice. Our three weeks of pre-experimental trials using a 10% (*v*/*v*) ethanol solution in adult C57BL/6J mice yielded approximately 10 g/kg/day of alcohol intake, based on the guidelines for converting animal doses to human equivalent doses by body surface area [17]. Consequently, we employed this condition to induce MetALD and investigated the role of 10% alcohol on metabolic phenotypes and gut microbiota/metabolites in LFD or HFD-fed mice, providing a comprehensive risk assessment of chronic moderate alcohol intake for populations with distinct dietary structures.

## 2. Materials and Methods

### 2.1. Animal Experiments

C57BL/6J male mice (weighing 20–22 g, SPF grade) were obtained from SPF Biotechnology Co., Ltd. (Beijing, China) and kept in a controlled barrier environment with consistent temperature, humidity, and lighting conditions (12 h light and 12 h dark). After one week of adaptation, mice were randomly divided into four groups (*n* = 10): (1) LFD group (10 kcal% fat, D12450J, Research Diets, New Brunswick, NJ, USA); (2) LFD+EtOH group (fed with 10% EtOH in drinking water; (3) HFD group (60 kcal% fat, D12492, Research Diets, USA); (4) HFD+EtOH (fed with 10% EtOH in drinking water). Body weight and food consumption were recorded weekly. One day prior to the endpoint, body fat mass was measured using quantitative magnetic resonance (QMR06-090H, Niumag, Suzhou, China). Fasting and non-fasting blood glucose levels were monitored via tail vein blood sampling with a glucose meter (ACCU-CHEK Performa, Roche Diabetes Care GmbH, Mannheim, Germany). Upon termination, mice were anesthetized. Blood samples and liver tissues were then collected. The liver samples were either snap-frozen in liquid nitrogen or fixed in 4% paraformaldehyde (#G1101, Servicebio, Wuhan, China) for subsequent histological analysis. Specifically, all investigators conducting sample collection, biochemical assays, histological analyses, and data statistics were blinded to group identities until all experimental measurements and preliminary data analysis were completed. The animal experiments were approved by the Institutional Animal Care and Use Committee of Chinese Academy of the Institute of Medicinal Biotechnology & Chinese Academy of Medical Sciences and conducted in accordance with the Guide for the Care and Use of Laboratory Animals. This section adheres to ARRIVE Guidelines 2.0 [18].

### 2.2. Health Monitoring and Exclusion Criteria

All mice were monitored daily for signs of distress and weekly for body weight. Pre-defined humane endpoints for exclusion from the study included (1) sustained body weight loss exceeding 20% of peak body weight; (2) severe lethargy, hunched posture, or inability to access food and water. Following the drinking of ethanol-containing water, one mouse in the LFD+EtOH group and two mice in the HFD+EtOH group met these criteria due to severe intolerance and were euthanized. Consequently, the final group sizes for all analyses were: LFD (*n* = 10), LFD+EtOH (*n* = 9), HFD (*n* = 10), HFD+EtOH (*n* = 8).

### 2.3. Analysis of Biochemical Parameters

Following the centrifugation of blood samples at a force of 2500× *g* for a duration of 10 min, the serum was carefully collected to facilitate subsequent biochemical analyses. The analysis involved measuring serum levels of various biomarkers, specifically alanine transaminase (ALT, C009-2-1), aspartate transaminase (AST, C010-2-1), triglycerides (TG, A110-1-1), and total cholesterol (CHO, A111-1-1). These measurements were conducted using commercially available assay kits sourced from the Nanjing Jiancheng Bioengineering Institute, located in Nanjing, China. Additionally, the concentration of insulin in the serum was assessed using a mouse insulin enzyme-linked immunosorbent assay (ELISA) kit (SEKM-0141, Solarbio, Beijing, China). To evaluate insulin resistance, the homeostasis model assessment-2 (HOMA2) index was employed, which can be calculated using the online tool available at the University of Oxford’s website (https://www.dtu.ox.ac.uk/homacalculator/ (accessed on 30 May 2025).). In terms of hepatic biochemistry analysis, liver tissues were homogenized to prepare for further analysis. Triglycerides and cholesterol levels were subsequently measured through the utilization of assay kits, adhering to the specific instructions provided by the manufacturers. To prepare liver homogenates, a lysis buffer was used, which was supplemented with a protease inhibitor cocktail (C0001, Targetmol, Boston, MA, USA) to protect the integrity of the proteins during the extraction process. Furthermore, the total protein concentration within these samples was quantified using a BCA protein assay kit (23225, Thermo Scientific, New York, NY, USA), ensuring accurate measurements for further investigation.

### 2.4. Histological Analysis

Hepatic histopathological features, including steatosis, ballooning degeneration, and inflammation, were evaluated by hematoxylin and eosin (H&E) staining. The severity was graded using the NAFLD Activity Score (NAS) system [19,20], which assesses steatosis (0–3), hepatocyte ballooning (0–2), and lobular inflammation (0–3). Liver fibrosis was examined by Masson’s trichrome staining, and the collagen area fraction was quantified using ImageJ software version 1.48. Lipid accumulation in the liver was further assessed by Oil Red O (ORO) staining on frozen sections. To evaluate hepatic inflammation, immunohistochemical (IHC) staining was performed using antibodies against the macrophage marker F4/80 (GB113373, Servicebio, Wuhan, China) and the neutrophil marker CD11b (GB11058, Servicebio, Wuhan, China).

### 2.5. 16S rRNA Gene Sequencing

Gut microbiota analysis was conducted on collected mouse fecal samples (*n* = 5) via 16S rRNA gene sequencing (Wuhan Metware Metabolism Biotechnology Co., Ltd., Wuhan, China). Following DNA extraction and purification from feces, the V3–V4 hypervariable region was amplified with modified primers. Libraries were constructed with a TruSeq DNA PCR-Free Sample Preparation Kit (20041794, Illumina, San Diego, CA, USA), while the library quality was evaluated using a Qubit 2.0 Fluorometer (Thermo Scientific) and an Agilent Bioanalyzer 2100 system. Sequencing was conducted on an Illumina NovaSeq platform. For statistical analysis, the nonparametric Kruskal–Wallis rank-sum test was employed to identify differentially abundant microbiota taxa among groups. Linear Discriminant Analysis Effect Size (LEfSe) was utilized to detect group-specific features. Random forest analysis (R package “varSelRF” version 4.3.2) was performed through bootstrap resampling to model group differences, with mean decrease accuracy used to assess variable importance. Spearman’s correlation-based hierarchical clustering of differential microbiota and metabolites was executed, and heatmaps were created with the Complex Heatmap package in R (version 4.3.2).

### 2.6. Targeted Metabolomics Analysis

Fecal samples of 5 mice in each group were used to conduct an analysis of gut metabolomics utilizing targeted sequencing of 700 metabolites (T700). Metabolomic analysis was performed using liquid chromatography–tandem mass spectrometry (LC-MS/MS). A custom Metware Database (MWDB) provided by Wuhan Metware Biotechnology Co. (Wuhan, China) supported the data analysis. Quantitative MS data were acquired in multiple reaction monitoring (MRM) mode on a QTRAP 6500+ triple quadrupole mass spectrometer (SCIEX, Framingham, MA, USA). Chromatographic peaks were processed and integrated using MultiQuant 3.0.3 software (SCIEX, USA), with reference to retention times and peak shapes from standard samples. Metabolite concentrations were determined by applying linear equations derived from standard curves to the integrated peak areas. The resulting dataset was subjected to extensive statistical evaluation, incorporating principal component analysis (PCA), orthogonal partial least squares discriminant analysis (OPLS-DA), and clustering analysis. Differentially identified metabolites were examined to reveal notable alterations in metabolic profiles.

### 2.7. RNA Extraction and Real-Time Quantitative PCR

Frozen liver tissues stored in liquid nitrogen were thawed on ice, and total RNA was extracted using the RaPure Total RNA Kit (R4011, Magen, Guangzhou, China). qRT-PCR was performed using the HiScript II One Step qRT-PCR SYBR Green Kit (Q221-01, Vazyme, Nanjing, China) with gene-specific primers listed in Table S2 [21,22]. The mRNA expression levels of target genes were normalized to glyceraldehyde-3-phosphate dehydrogenase (Gapdh), and calculated using the ΔΔCt method.

### 2.8. Statistical Analyses

The results are shown as mean ± standard deviation (SD) or as representative figures. The Shapiro–Wilk test was used to assess normality. For datasets that met the normality assumption, a one-way analysis of variance (ANOVA) was conducted. When the ANOVA indicated a significant difference, the Student–Newman–Keuls (SNK) post hoc test was employed to control for Type I errors across multiple pairwise comparisons. For data that violated the normality assumption, the nonparametric Kruskal–Wallis H test was used, followed by Dunn’s post hoc test for pairwise comparisons. All analyses were conducted using GraphPad Prism 8 software. Statistical significance was defined as * *p* < 0.05 and ** *p* < 0.01.

## 3. Results and Discussion

### 3.1. The Effect of Moderate Alcohol Intake on Basic Phenotypes in LFD or HFD-Fed Mice

**Figure 1:**
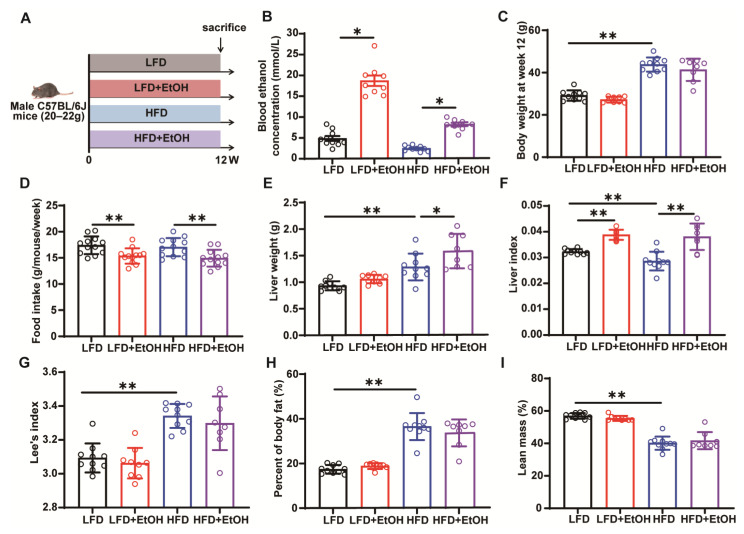
The effect of moderate alcohol intake on basic phenotypes in LFD or HFD-fed mice. (**A**) Animal experimental schedule. (**B**) The concentration of blood alcohol. (**C**) Body weight at week 12. (**D**) Average food intake per week. (**E**) Liver weight. (**F**) Liver index. (**G**) Lee’s index. (**H**) Body fat. (**I**) Lean mass. *n* = 8~10, * *p* < 0.05 and ** *p* < 0.01. LFD, low-fat diet; HFD, high-fat diet.

To establish the MetALD mice model and investigate the effects of chronic moderate alcohol intake on basic metabolic phenotypes, mice were fed with LFD or HFD for 12 weeks, with or without the addition of 10% alcohol (*v*/*v*, EtOH) in their drinking water (Figure 1A). Notably, after 8 weeks of alcohol consumption, one mouse (10%) in the LFD+EtOH group and two mice (20%) in the HFD+EtOH group exhibited intolerance, resulting in sudden weight loss and subsequent exclusion from the study. By week 12, elevated blood alcohol levels in both LFD and HFD-fed mice confirmed successful alcohol consumption (Figure 1B). Predictably, the HFD group showed a significantly increased body weight, while alcohol intake slightly decreased the body weight in both LFD and HFD-fed mice, although with no statistically significant difference (Figure 1C). This may be attributed to the reduction in food intake observed in the corresponding groups throughout the experiment (Figure 1D). Compared to the LFD or HFD groups, alcohol consumption significantly elevated liver weight (Figure 1E) and index (Figure 1F). Additionally, the HFD resulted in a high Lee’s index, increased body fat, and decreased lean mass, whereas alcohol intake did not induce significant changes in these indicators in either LFD or HFD-fed mice (Figure 1G–I). Overall, following chronic moderate alcohol exposure, the mouse model partially reproduces features consistent with MetALD, exhibiting moderate changes in basic metabolic phenotypes, a slight decrease in body weight and food intake, alongside an increase in liver weight and liver index.

### 3.2. The Effect of Moderate Alcohol Intake on Hyperlipidemia and Insulin Resistance in LFD or HFD-Fed Mice

**Figure 2:**
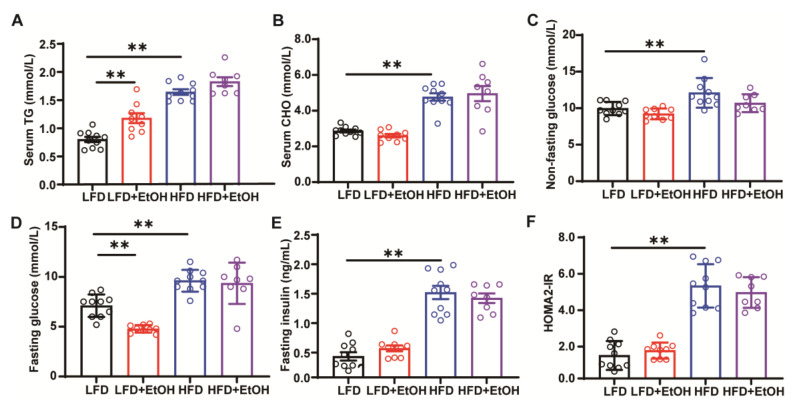
The effect of moderate alcohol intake on hyperlipidemia and insulin resistance in LFD or HFD-fed mice. (**A**) Serum TG. (**B**) Serum CHO. (**C**) Non-fasting blood glucose. (**D**) Fasting blood glucose. (**E**) Fasting insulin. (**F**) HOMA2-IR index. *n* = 8~10, ** *p* < 0.01. CHO, cholesterol; HFD, high-fat diet. LFD, low-fat diet; TG, triglycerides.

MetALD is usually accompanied by hyperlipidemia and insulin resistance. In this study, feeding a high-fat diet significantly increased serum TG, serum CHO, non-fasting and fasting blood glucose, fasting insulin, and the insulin resistance index HOMA2-IR compared to the LFD group (Figure 2A–F). Overall, moderate alcohol consumption in HFD-fed mice did not result in significant changes in these indicators (Figure 2A–F), suggesting that a high-fat diet might potentially obscure the effects of moderate alcohol intake. Notably, although moderate alcohol had no evident impact on serum CHO (Figure 2B), non-fasting blood glucose (Figure 2C), fasting insulin (Figure 2E), and HOMA2-IR index (Figure 2F), we did observe that it induced higher serum TG levels (Figure 2A) and lower fasting glucose levels (Figure 2D). These findings highlight that dysfunctional glucose and lipid metabolism are principally attributed to chronic, moderate alcohol consumption in lean individuals who consume alcohol exclusively. In contrast, in obese individuals, it is primarily driven by a high-energy diet.

### 3.3. The Effects of Moderate Alcohol Intake on Hepatic Steatohepatitis and Liver Injury

**Figure 3:**
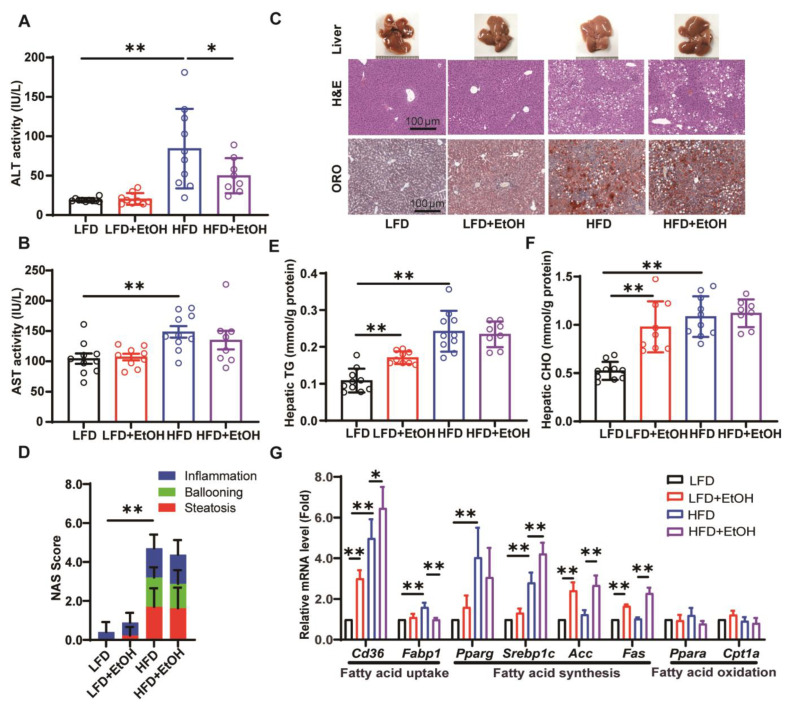
The effects of moderate alcohol intake on hepatic steatohepatitis and liver injury. (**A**) Serum ALT. (**B**) Serum AST. (**C**) Representative liver appearance, H&E staining, and oil red O (ORO) staining of liver sections. (**D**) Quantification of NAFLD Activity Score (NAS). (**E**) Liver TG. (**F**) Liver CHO. (**G**) qRT-PCR analysis of *Cd36*, *Fabp1*, *Pparg*, *Srebp1c*, *Acc*, *Fas*, *Ppar*α, *Cpt1a* mRNA levels in the livers of mice. *n* = 8~10, * *p* < 0.05 and ** *p* < 0.01. CHO, cholesterol; HFD, high-fat diet. TG, triglycerides; LFD, low-fat diet.

The effect of moderate alcohol intake on the severity of hepatic steatohepatitis and liver injury in mice fed with LFD or HFD was further evaluated. The HFD significantly raised serum ALT (Figure 3A) and AST (Figure 3B) levels, indicating aggravated liver injury. Conversely, alcohol intake resulted in a significant decrease in serum ALT levels (Figure 3A) and a relevant decrease in AST levels (Figure 3B) in HFD-fed mice, while LFD-fed mice showed no apparent changes. According to previous reports, this may be because moderate alcohol consumption reduces hepatic apoptosis, thereby lowering ALT levels [7,23]. The observed decrease in serum ALT levels in alcohol-fed groups, despite the presence of steatosis, underscores the complex and sometimes non-linear relationship between alcohol consumption and conventional liver injury biomarkers. This pattern, noted in some studies of early-stage metabolic liver disease [7,23,24], suggests that ALT alone may not fully capture the underlying hepatic stress in this specific context, highlighting the importance of correlating biochemical data with histological findings. Histopathologically, mice in the LFD+EtOH group exhibited an increase in liver stiffness, and slight hepatic steatosis and inflammation compared to the LFD group (Figure 3C). The HFD group exhibited marked liver enlargement, steatosis, inflammation, and ballooning; however, alcohol had minimal impact on these parameters in HFD-fed mice (Figure 3C). The NAFLD Activity Score (NAS) further corroborated these findings (Figure 3D). Hepatic TG and total CHO levels in HFD-fed mice were elevated compared to those in LFD-fed mice, and alcohol significantly increased them in the LFD but not HFD-fed mice (Figure 3E,F). This further verifies the conclusion that a high-fat diet, but not moderate alcohol consumption, is the primary factor contributing to dysfunction in glucose and lipid metabolism. Regarding metabolism-related gene expression, the results indicated that alcohol enhanced the expression of lipid uptake-related gene *Cd36* and lipid synthesis-related genes *Acc* and *Fas* in both the LFD and HFD-fed mice, while having minimal effect on genes related to fatty acid oxidation (Figure 3G). Meanwhile, alcohol also enhanced the mRNA level of *Srebp1c* but decreased the *Fabp1* in HFD-fed mice. These changes may explain the mild steatosis in the LFD+EtOH group, while the lack of more severe steatosis in the HFD+EtOH group is possibly because the HFD itself alters the expression of lipid metabolism-related genes, thereby masking the effects of alcohol. In summary, we observed that moderate alcohol intake slightly induced hepatic steatosis and inflammation in LFD-fed mice with higher expression of lipid uptake and lipid synthesis-related genes. However, it did not obviously worsen hepatic steatohepatitis in HFD-fed mice but exhibited a decrease in transaminase levels.

### 3.4. The Effects of Moderate Alcohol Intake on Inflammation and Fibrosis

**Figure 4:**
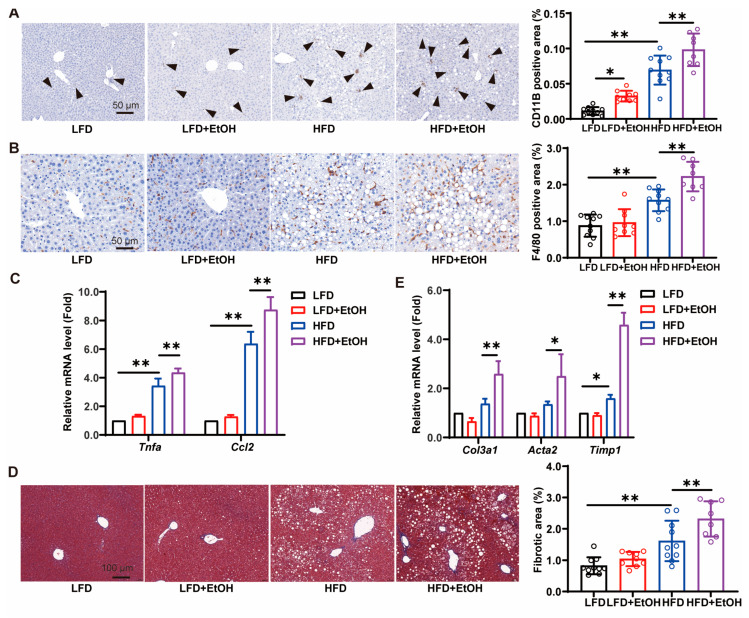
The effects of alcohol intake on inflammation and fibrosis. (**A**) CD11b immunohistochemical stains and qualification of liver sections. (**B**) F4/80 immunohistochemical stains and qualification of liver sections. (**C**) qRT-PCR analysis of *Tnfa* and *Ccl2* mRNA levels in the liver of mice. (**D**) Masson staining and fibrotic area qualification of liver sections. (**E**) qRT-PCR analysis of *Col3a1*, *Acta2*, and *Timp1* mRNA levels in the livers of mice. Black arrow represents the CD11B positive cells. *n* = 8~10, * *p* < 0.05 and ** *p* < 0.01. HFD, high-fat diet; LFD, low-fat diet.

Excessive accumulation of dietary lipids within hepatocytes induces hepatic steatosis, oxidative stress, and inflammatory cascades [22,25]. To evaluate the inflammatory status, we assessed the infiltration of neutrophils and macrophages in the liver by immunohistochemical staining and inflammatory gene expression by qRT-PCR. Compared to the LFD group, the protein expression of neutrophil marker CD11b (Figure 4A), macrophage marker F4/80 (Figure 4B), as well as the hepatic mRNA expression of inflammatory gene *Tnfα* and *Ccl2* (Figure 4C) in the HFD group were significantly increased, suggesting a state of liver inflammation. Moderate alcohol consumption appeared to amplify the inflammation in HFD-fed mice, but merely exhibited a worsened tendency in LFD-fed mice (Figure 4A–C). Severe MetALD is accompanied by fibrosis, elevating the risk of hepatocellular carcinoma. Masson staining of liver tissues revealed that the HFD induced more severe liver fibrosis than the LFD, and alcohol significantly increased the fibrotic area, thus worsening the degree of liver fibrosis in HFD-fed mice (Figure 4D). The significant upregulation of pro-fibrotic genes, such as *Col3a1*, *Acta2*, and *Timp1*, in the HFD+EtOH group further verified its role (Figure 4E). However, we did not observe the evident fibrosis state in the LFD-fed mice after moderate alcohol intake for 12 weeks (Figure 4D,E). Collectively, these findings suggest that moderate alcohol intake exacerbates liver inflammation and fibrosis in HFD-fed mice while having minimal effect in LFD-fed mice, likely due to combined nutritional and alcohol-related drivers or better tolerance in healthy mice.

### 3.5. The Effects of Moderate Alcohol Intake on Gut Microbiota

**Figure 5:**
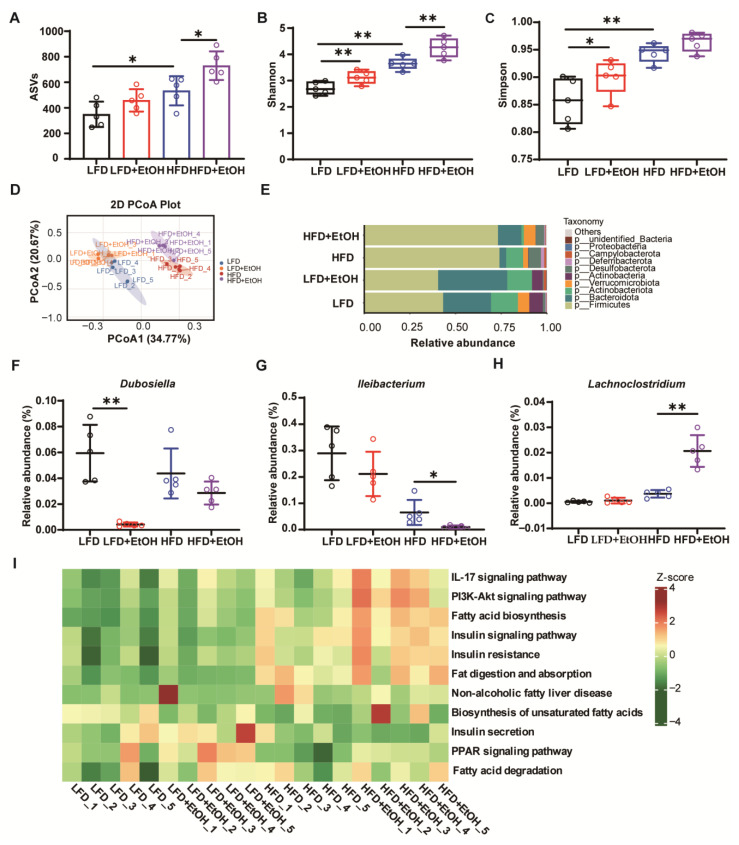
The effects of moderate alcohol intake on gut microbiota. (**A**) Amplicon Sequence Variants (ASVs). (**B**) Shannon index. (**C**) Simpson index. (**D**) Principal coordinates analysis (PCoA) plot. (**E**) Relative abundance at the phylum level. (**F**–**H**) Relative abundance of *Dubosiella*, *Ileibacterium*, and *Lachnoclostridium* genera. (**I**) Prediction of inflammation and metabolism-related microbiome function based on the KEGG database. *n* = 8~10, * *p* < 0.05 and ** *p* < 0.01. HFD, high-fat diet; LFD, low-fat diet.

Gut microbiota usually maintains host homeostasis through its metabolites that continuously shape the host’s immune system and metabolic processes [26]. Therefore, we assessed the effects of moderate alcohol intake on gut microbiota using 16S rRNA sequencing. ASV (Amplicon Sequence Variant)-based analysis revealed that moderate alcohol consumption led to an increase in the number of ASVs in both LFD- and HFD-fed mice (Figure 5A), consistent with the upregulation of microbial α-diversity observed by the Shannon index (Figure 5B) and Simpson index (Figure 5C). Principal coordinates analysis (PCoA) demonstrated that the HFD evidently altered the gut microbiota community structure, while alcohol partially influenced it in both the LFD and HFD-fed mice (Figure 5D). At the phylum level, *Bacteroidota* was one of the dominant bacterial groups present in all groups, which has been reported to produce short-chain fatty acids to maintain intestinal health [27] and is negatively correlated with the degree of obesity [28,29]. In our study, the relative ratio of *Bacteroidota* clearly declined in the HFD group, while alcohol was observed to increase it in both LFD and particularly HFD-fed mice (Figure 5E). This alteration may partially explain the slightly decreased body weight after alcohol intake. Further analysis at the genus level revealed specific changes in gut microbiota. For example, the relative abundance of *Dubosiella* and *Ileibacterium*, both of which could produce short-chain fatty acids (SCFAs), was reported to decline in metabolic diseases [30,31,32,33], and all of them were decreased following alcohol intake in LFD and HFD-fed mice (Figure 5F,G). In contrast, *Lachnoclostridium*, which could produce trimethylamine to promote atherosclerosis [34], was further increased after alcohol consumption compared to the HFD group (Figure 5H). These changes may partially explain the association between inflammatory/fibrotic phenotypes and alcohol intake, particularly in the HFD+EtOH group. KEGG analysis also indicated that pathways related to inflammation, insulin signaling, and lipid metabolism were enriched in the HFD+EtOH group (Figure 5I). These findings suggest that moderate alcohol intake significantly alters the gut microbiota composition in both HFD and LFD groups, and specifically exacerbates inflammation and metabolic-related pathways when combined with HFD.

### 3.6. The Effect of Moderate Alcohol Intake on Gut Metabolites

**Figure 6:**
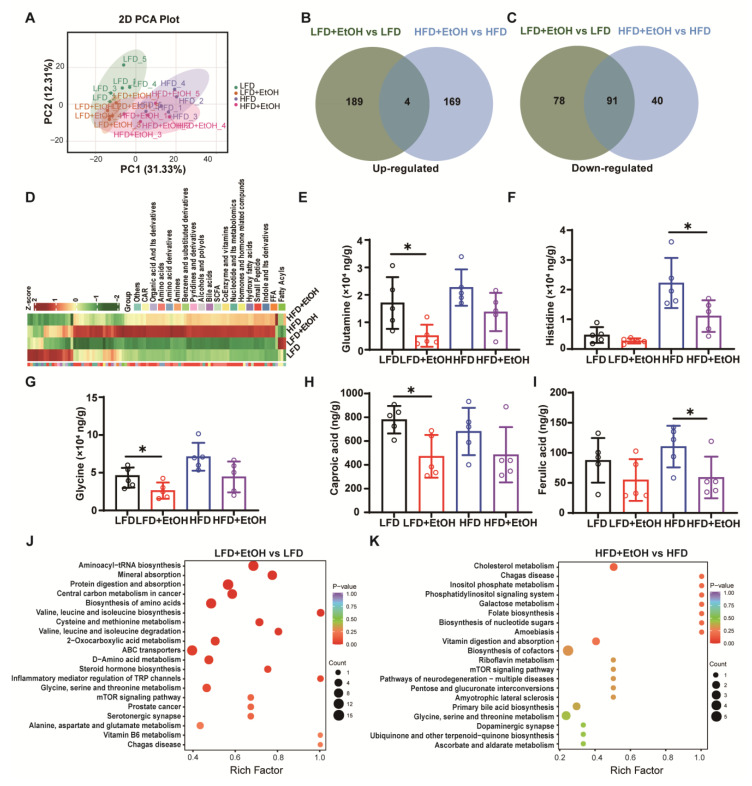
The effect of moderate alcohol intake on gut metabolites. (**A**) Principal component analysis (PCA) plot. (**B**,**C**) Venn plots showing commonly upregulated or downregulated metabolites between LFD+EtOH vs. LFD and HFD+EtOH vs. HFD groups. (**D**) Cluster heatmap of differential metabolites. (**E**–**I**) Content of glutamine, histidine, glycine, caproic acid, and ferulic acid. (**J**,**K**) KEGG pathway enrichment analysis of differential metabolites. *n* = 8~10, * *p* < 0.05. HFD, high-fat diet; LFD, low-fat diet.

To further explore the effect of moderate alcohol intake on gut microbiota, we further analyzed the metabolites by targeted metabolomics methods. A total of 306 metabolites were detected, and PCA analysis showed that the HFD evidently altered gut metabolites, while alcohol partially changed them in both LFD and HFD-fed mice (Figure 6A). We set the fold change (FC) threshold of less than 0.75 or greater than 1.5 as the criterion for the screening of changed metabolites. The results showed that alcohol led to a total of 95 commonly altered metabolites in LFD and HFD-fed mice, including amino acids and their derivatives, short-chain fatty acids, and other metabolites (Table S1). Among these changed metabolites, 4 were commonly upregulated (Figure 6B), and 91 were downregulated (Figure 6C). The cluster heatmap clearly distinguished these shared altered metabolites (Figure 6D), indicating that alcohol consumption lowered most gut metabolite levels. Among the 95 commonly changed metabolites, glutamine was observed to decrease after alcohol intake (Figure 6E), both in LFD and HFD-fed mice. Previous studies have shown that glutamine can mediate anti-inflammatory effects via reduction in nuclear protein O-GlcNAcylation [35] and activating the Nrf2 pathway [36], and its decline may partially explain the varying degrees of inflammation exacerbation by alcohol in both LFD and HFD-fed mice. Additionally, the higher glutamine levels in HFD-fed mice—which enhance alcohol metabolism [37,38]—likely explain their lower blood alcohol concentrations compared to LFD-fed mice.

Moreover, histidine, a semi-essential amino acid, exhibits anti-inflammatory properties, whereas glycine scavenges free radicals produced during alcohol metabolism, thereby attenuating lipid peroxidation and hepatocyte damage [39]. SCFAs such as caproic acid reduce hepatic triglycerides, enhance insulin sensitivity, and suppress lipid synthesis genes [40], while ferulic acid inhibits lipid synthesis and exhibits anti-inflammatory effects [41,42]. All of the metabolites mentioned above were observed to decline or show a decreasing trend following alcohol consumption in LFD and HFD-fed mice (Figure 6F–I). These changes may contribute to the induction of lipid synthesis-related gene expression, exacerbation of inflammation, and fibrosis resulting from alcohol intake (Figure 3 and Figure 4). KEGG enrichment analysis of differential metabolites revealed that alcohol primarily affected amino acid metabolism in the LFD group (Figure 6J), whereas it predominantly influenced cholesterol metabolism in the HFD group (Figure 6K). This finding further suggests that moderate alcohol intake may exert a distinct physiological role in individuals with different dietary backgrounds, nutrition, or metabolic states.

## 4. Discussion

**Figure 7:**
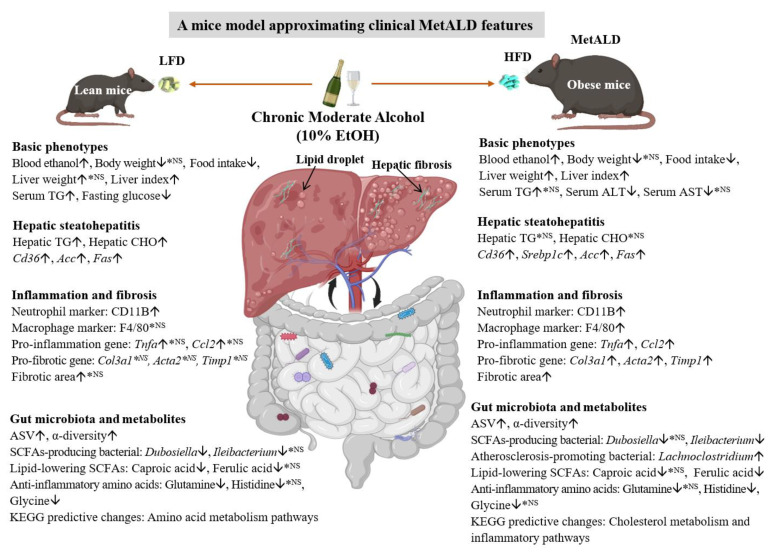
Comparative evaluation of chronic moderate alcohol intake on metabolic phenotypes and gut microbiota/metabolites in lean and obese mice fed with distinct dietary structures. ↑: upregulation; ↓: downregulation; ^*NS^: no significant change; ALT: alanine aminotransferase; ASV: Amplicon Sequence Variant; AST: aspartate aminotransferase; CHO: cholesterol; HFD: high-fat diet; KEGG: Kyoto Encyclopedia of Genes and Genomes; LFD: low-fat diet; MetALD: metabolic and alcohol-associated liver disease; SCFAs: short-chain fatty acids; TG: triglyceride.

The updated conceptual framework, from NAFLD to MASLD, centers on a deeper understanding of diverse subpopulations and specific health risks [43,44,45]. While alcohol abuse is a well-admitted risk factor for fatty liver disease, the implications of chronic moderate alcohol intake—particularly under the newly proposed concept of MetALD—remain insufficiently clarified [5]. This study systematically evaluated the disease risks associated with chronic moderate alcohol consumption in mice across various dietary backgrounds, and established a translational MetALD mice model for preclinical evaluation (Figure 7). A key novel aspect of our approach is the integration of metabolomic and microbiome data, which provides multi-optic insight into how moderate alcohol remodels the gut-liver axis. We observed that moderate alcohol in LFD-fed mice caused only mild dyslipidemia and hepatic steatosis, without significant inflammatory or fibrotic worsening. Notably, in HFD-fed mice, alcohol did not exacerbate systemic glucose or lipid metabolism but markedly enhanced liver inflammation and fibrosis. Furthermore, long-term moderate alcohol intake markedly reshaped the gut microbial ecosystem, enhancing microbial diversity while downregulating some beneficial metabolites such as short-chain fatty acids. These microbiota/metabolites alterations were associated with an increased risk of worsened metabolic, inflammation, and pro-fibrotic outcomes, particularly under high-fat conditions. The main outcomes of the experimental model focusing on alcohol-treated lean and obese mice are summarized in Table S3.

Alcohol is a non-metabolic cause of fatty liver, and it also interacts with dietary factors for the progression or prognosis of multiple liver diseases [46,47,48]. Actually, the risk of moderate alcohol intake for fatty liver differs from that of extreme alcohol abuse. In this study, to avoid the mortality and stress of alcohol binges by gavage, the mice were given HFD and free alcohol solution (10% *v*/*v*) in drinking water (approximately 50 g/kg/day for males based on our pre-experimental trials) to establish a MetALD model, which simulated the clinical unbalanced diet nutrition and consumption of alcohol in MetALD patients (20–50 g/day for females and 30–60 g/day for males) [5]. It is noteworthy that, compared to the LFD-fed mice, the alcohol levels in the HFD-fed mice were slightly lower (Figure 1B), which aligns with previous reports regarding alcohol detection [49]. The model captures the core metabolic disturbances of MetALD, as the high-fat diet robustly induced hyperlipidemia and insulin resistance (Figure 2), mirroring the characteristic metabolic dysfunction in patients. The model also captures the specific impact of alcohol on lipid metabolism, as alcohol consumption significantly elevated serum TG and exacerbated hepatic steatosis in LFD-fed mice (Figure 2A and Figure 3), consistent with the known effect of alcohol to directly promote lipid metabolic disorders in the clinical MetALD phenotype. Generally, alcohol consumption exacerbates liver cell damage and leads to elevated serum transaminases. However, we did observe a slightly decreased serum ALT in the HFD+EtOH group and no changes in other groups after moderate alcohol intake (Figure 3A), which is consistent with previous reports [7,50]. We speculate that this may be related to the ratio of alcohol and HFD consumption, whereas long-term HFD intake might be more likely to induce liver injury than moderate alcohol intake. The pathogenesis of both MASLD and ALD is related to immune cell infiltration and innate immune response [51,52]. We also observed increased macrophage and neutrophil infiltration in the alcohol-treated mice (Figure 4A,B), confirming that alcohol can exacerbate activation of Kupffer cells and subsequent secretion of cytokines [53]. Regarding liver fibrosis, the administration of 10% alcohol in LFD-fed mice did not result in significant fibrosis. However, the combination of HFD with moderate alcohol intake led to notable fibrotic gene expression and histological alterations (Figure 4D,E), confirming the possibility of fibrosis induction within 12 weeks by the dual driver of diets and alcohol.

The gut microbiome plays key roles in metabolic diseases [54,55]. The fecal microbiota composition and biodiversity of HFD-fed mice were significantly increased compared with LFD-fed mice, aligning with previously reported data [56,57]. Additionally, these indicators further increased in the alcohol group, consistent with the findings that human alcohol drinkers had significantly higher gut microbial biodiversity than non-drinkers [58]. However, drinking patterns and sex influenced how ethanol consumption shaped the gut microbiome in mice [59,60]. Further analyses at the genus level revealed that certain bacteria, such as *Dubosiella* and *Ileibacterium*, whose abundances decreased in metabolic disease [32,33], also showed reductions following alcohol intake (Figure 5F,G). Conversely, *Lachnoclostridium*, associated with arteriosclerosis promotion [34], increased in the alcohol intake group (Figure 5H). Nevertheless, the apparent minimal impact of moderate alcohol consumption on metabolic parameters is likely masked by the more dominant effects of the high-fat diet. The functional enrichment of inflammatory pathways in the HFD group may be associated with alcohol’s alteration of the gut microbiota and its secondary metabolites, like bile acids [61]. However, this correlation also needs to be interpreted with caution. Accumulating evidence suggests that changes in the microbial community may not be an initial driver of disease, but rather an adaptive reorganization following an altered physiological environment in the host [62,63].

MetALD is frequently associated with host metabolic disorders, including lipid peroxidation, glucose metabolism, and amino acid metabolism. In this study, a widely targeted metabolomics analysis showed that ethanol exposure reduced several key metabolites: (1) Glutamine levels decreased following alcohol consumption (Figure 6E), which may exacerbate the inflammatory response [35,36]. (2) Anti-inflammatory amino acids, such as histidine and glycine (Figure 6F,G), showed a decline, indicating heightened inflammatory response [39]. (3) The lipid-lowering SCFA caproic acid was also reduced (Figure 6H), promoting lipid accumulation [40]. These alterations in metabolites imply an association between alcohol intake and the exacerbation of inflammation and fibrosis. However, the effects of alcohol on metabolic indicators are not as pronounced as its impact on promoting inflammation and fibrosis. This discrepancy may arise from the regulation of metabolic indicators by various factors, including diet and hormones, which can buffer short-term fluctuations through compensatory mechanisms. Consequently, changes in metabolic indicators tend to be more gradual, whereas inflammation and fibrosis exhibit progressive characteristics. Additionally, differences in the sensitivity of detection methods may contribute to these observations.

A key limitation of this study is that alcohol intake may have slightly reduced food consumption. Due to the absence of a pair-feeding design, we cannot dissociate the specific metabolic effects of alcohol from the confounding effects of reduced caloric intake, which may potentially impact the experimental results. However, this is generally expected, as indicated by other reports [64]. We also assumed that the moderate and long-term patterns of alcohol consumption may predict pathological drinking behaviors, which can induce extensive physiological disturbances and metabolic toxicity, ultimately leading to a net reduction in food intake [65]. Furthermore, reduced ghrelin levels in chronic alcoholics are associated with decreased food intake and suppressed energy metabolism [66,67]. Notably, translational limitations include interspecies differences (mouse vs. human), specific experimental doses, and the absence of female mice, restricting generalizability. Additionally, we have not yet identified the specific reason for moderate alcohol intake leading to intolerance in 10–20% of the mice, and lower fasting blood glucose levels in LFD-fed mice. The reason that lower blood alcohol levels were observed in the HFD+EtOH group compared to the LFD+EtOH group remains to be clarified. Relationally, one possible explanation for this relationship is that HFD accelerated alcohol metabolism through complex mechanisms, such as by promoting PPARα expression [68]. Furthermore, we acknowledge that our multi-omics findings are correlative in nature. To establish causality in future research, it will be essential to conduct direct functional studies, such as fecal microbiota transplantation or supplementation with specific metabolites. Despite the ongoing inconsistencies in understanding how alcohol exacerbates disease severity, it is certain that drinking patterns, for example, chronic plus binge, moderate, low ethanol intake, or even chronic vapour alcohol exposure, and gender can affect the impact of ethanol consumption on the disease progression [59,60]. At least, the model established under experimental conditions in our study provides valuable data and references for MetALD research.

## 5. Conclusions

In summary, the findings from our research indicate that the interplay between moderate alcohol intake and dietary fat content is a pivotal determinant of metabolic and inflammatory outcomes in the liver. This interaction suggests that the health implications of alcohol cannot be viewed in isolation but must be considered within the broader context of an individual’s overall diet. Our findings warrant further mechanistic exploration to unravel the precise molecular pathways through which diet and alcohol converge to either protect or harm the liver.

## Data Availability

The data presented in this study are available on request from the corresponding author.

## Supplementary Materials

The following supporting information can be downloaded at: https://www.mdpi.com/article/10.3390/nu17233658/s1, Table S1: The commonly altered metabolites in LFD and HFD-fed mice with alcohol treatment; Table S2: Primers for the quantification of inflammatory cytokines, lipid metabolism-related genes, and liver fibrosis-related genes in mice; Table S3: Comparison of main outcomes between alcohol-treated lean and obese mice.

## Abbreviations

The following abbreviations are used in this manuscript:

ALD	Alcohol-associated liver disease
ALT	Alanine aminotransferase
AST	Aspartate aminotransferase
ASV	Amplicon Sequence Variant
CHO	Cholesterol
EtOH	Ethanol
HFD	High-fat diet
HOMA2-IR	Homeostatic Model Assessment 2 of Insulin Resistance
KEGG	Kyoto Encyclopedia of Genes and Genomes
LFD	Low-fat diet
MASLD	Metabolic dysfunction-associated steatotic liver disease
MetALD	Metabolic and alcohol-associated liver disease
NAFLD	Nonalcoholic fatty liver disease
NAS	NAFLD activity score
PCA	Principal component analysis
PCoA	Principal coordinates analysis
SCFA	Short-chain fatty acid
SLD	Steatotic liver disease
TG	Triglyceride
