# Supplementary material for: Effects of Chronic Moderate Alcohol Intake on Metabolic Phenotypes and Gut Microbiota in Lean and Obese Mice with Distinct Dietary Structures
Source: Nutrients. 2025 Nov 23;17(23):3658. doi: 10.3390/nu17233658 (PMC12693642; doi:10.3390/nu17233658)
Supplement: Supplementary file 1 [file nutrients-17-03658-s001.zip › nutrients-3956400-supplementary.pdf]

Supplementary Information

Effects of chronic moderate alcohol intake on metabolic phenotypes and gut microbiota in lean and obese mice with distinct dietary structures

Jiu-Jiao Gao[1†], Zi-Die Nian[1†], Ning Li [1], Tong Wang[1], Han Sun[1], Mei Tang[1], Jian-Rui Li[1], Biao Dong [1,2], Jing-Chen Xu [1], Yue Gong [1], Xin-Yue Liu[1], Jian-Dong Jiang [1,2,3], Hu Li [1,2,3*], Zong-Gen Peng [1,2,3*]

1 CAMS Key Laboratory of Antiviral Drug Research, Institute of Medicinal Biotechnology, Chinese Academy of Medical Sciences & Peking Union Medical College, Beijing 100050, China. j2024080004@pumc.edu.cn (J.-J.G.); s2023010018@pumc.edu.cn (Z.-D.N.); s2024010018@student.pumc.edu.cn (N.L.); b2024010032@student.pumc.edu.cn (T.W); b2023010026@pumc.edu.cn (H.S.); tangmei@imb.pumc.edu.cn (M.T.); lijianrui@imb.pumc.edu.cn (J.-R.L.); dongbiao@imb.cams.cn (B.D.); xujingchen@imb.pumc.edu.cn. (J.-C.X.); gongyue@imb.pumc.edu.cn. (Y.G.); s2023010043@pumc.edu.cn(X.-Y.L.); jiangjiandong@imb.cams.cn. (J.-D.J.).

2 Key Laboratory of Biotechnology of Antibiotics, The National Health and Family Planning Commission (NHFPC), Institute of Medicinal Biotechnology, Chinese Academy of Medical Sciences & Peking Union Medical College, Beijing 100050, China

3 State Key Laboratory of Bioactive Substance and Function of Natural Medicines, Institute of Medicinal Biotechnology, Chinese Academy of Medical Sciences & Peking Union Medical College, Beijing 100050, China

* Correspondence: lihu0112@imb.pumc.edu.cn (H.L.); pengzonggen@imb.pumc.edu.cn (Z.-G.P.); Tel.: +86-10-63165281 (H.L.), +86-10-63166129 (Z.-G.P.).

†These authors contributed equally to this work

Contents

Table S1. The commonly altered metabolites in LFD and HFD-fed mice with alcohol treatment

Table S2. Primers for the quantification of inflammatory cytokines, lipid metabolism-related genes, and liver fibrosis-related genes in mice

Table S3. Comparison of main outcomes between alcohol-treated lean and obese mice

Table S1. The commonly altered metabolites in LFD and HFD-fed mice with alcohol treatment.

Compounds	Class	Fold change (LFD+EtOH/LFD)	Fold change (HFD+EtOH/HFD)	Changes*
Betaine	Others	2.683	1.806	up
Isobutyryl-carnitine	Acyl carnitines	2.207	1.515	up
Isovaleryl-carnitine	Acyl carnitines	1.580	1.693	up
Ureidopropionic acid	Organic acid And Its derivatives	1.997	3.085	up
Gln	Amino acids	0.314	0.606	down
Thr	Amino acids	0.435	0.581	down
Ala	Amino acids	0.528	0.681	down
His	Amino acids	0.625	0.499	down
Met	Amino acids	0.521	0.686	down
Tyr	Amino acids	0.544	0.708	down
Val	Amino acids	0.493	0.688	down
Arg	Amino acids	0.187	0.395	down
Asn	Amino acids	0.262	0.624	down
Trp	Amino acids	0.501	0.563	down
Phe	Amino acids	0.513	0.708	down
Cys	Amino acids	0.251	0.563	down
Ile	Amino acids	0.493	0.609	down
Ser	Amino acids	0.463	0.537	down
Asp	Amino acids	0.493	0.653	down
Leu	Amino acids	0.501	0.638	down
Lys	Amino acids	0.465	0.641	down
Gly	Amino acids	0.575	0.625	down
Orn	Amino acids	0.461	0.490	down
N,N-dimethylarginine	Amino acid derivatives	0.535	0.716	down
N-Acetylproline	Amino acid derivatives	0.353	0.584	down
H-Lys(Me)-OH	Amino acid derivatives	0.734	0.705	down
Sphinganine	Amines	0.648	0.652	down
2-aminobenzoic acid	Benzene and substituted derivatives	0.693	0.629	down
4-Pyridoxic acid	Pyridines and derivatives	0.621	0.736	down
Myo-Inositol	Alcohols and polyols	0.536	0.469	down
Dehydrolithocholic acid	Bile acids	0.388	0.546	down
Lithocholic acid-3-sulfate	Bile acids	0.211	0.356	down
Valeric Acid	Short-chain fatty acids	0.736	0.690	down
Caproic Acid	Short-chain fatty acids	0.673	0.712	down
Biotin	CoEnzyme and vitamins	0.479	0.651	down
5'-Deoxy-5'-methylthioadensine	Nucleotide and Its metabolomics	0.389	0.747	down
S-adenosyl-L-methioninate	Nucleotide and Its metabolomics	0.584	0.612	down

Compounds	Class	Fold change (LFD+EtOH/LFD)	Fold change (HFD+EtOH/HFD)	Changes*
N6-methyladenosine	Nucleotide and Its metabolomics	0.656	0.723	down
Cytidine	Nucleotide and Its metabolomics	0.637	0.651	down
Uridine	Nucleotide and Its metabolomics	0.479	0.577	down
Adenosine	Nucleotide and Its metabolomics	0.695	0.703	down
2'-deoxycytidine	Nucleotide and Its metabolomics	0.655	0.666	down
2'-deoxyguanosine	Nucleotide and Its metabolomics	0.494	0.623	down
Guanosine	Nucleotide and Its metabolomics	0.528	0.465	down
Inosine	Nucleotide and Its metabolomics	0.357	0.628	down
Guanine	Nucleotide and Its metabolomics	0.505	0.654	down
2-Methoxy Estrone	Steroids and steroid derivatives	0.477	0.727	down
2-Hydroxyhexadecanoic acid	Fatty Acyls	0.573	0.720	down
Palmitoyl-carnitine	Acyl carnitines	0.749	0.721	down
Gly-Leu	Small Peptide	0.389	0.732	down
Ala-Gln	Small Peptide	0.663	0.743	down
Ala-His	Small Peptide	0.456	0.575	down
Ile-Ile	Small Peptide	0.164	0.703	down
Ser-Glu	Small Peptide	0.554	0.657	down
Gly-Ser	Small Peptide	0.480	0.670	down
His-Ser	Small Peptide	0.422	0.750	down
His-Tyr	Small Peptide	0.305	0.540	down
Met-Tyr	Small Peptide	0.309	0.725	down
Gly-Tyr	Small Peptide	0.375	0.624	down
Ala-Pro	Small Peptide	0.432	0.633	down
Ala-Glu	Small Peptide	0.353	0.483	down
Val-Ala	Small Peptide	0.259	0.688	down
Val-Val	Small Peptide	0.183	0.621	down
Trp-Glu	Small Peptide	0.348	0.612	down
Gly-Gly	Small Peptide	0.491	0.592	down
Val-Pro-Leu	Small Peptide	0.313	0.699	down
γ-Glu-Phe	Small Peptide	0.557	0.647	down
Phe-Asn	Small Peptide	0.266	0.574	down
Phe-Val	Small Peptide	0.234	0.693	down
Val-Ile	Small Peptide	0.161	0.559	down
Ile-Trp	Small Peptide	0.313	0.666	down
Val-Tyr	Small Peptide	0.233	0.502	down
Ile-Pro-Ile	Small Peptide	0.236	0.503	down
Val-Met	Small Peptide	0.331	0.625	down
Gly-Thr	Small Peptide	0.480	0.691	down
His-Leu	Small Peptide	0.283	0.576	down
Phe-Tyr	Small Peptide	0.256	0.323	down
Phe-Gly	Small Peptide	0.235	0.479	down
Gly-Glu	Small Peptide	0.515	0.689	down
Glu-Val	Small Peptide	0.158	0.629	down
Tyr-Tyr	Small Peptide	0.110	0.624	down
Tyr-Ala	Small Peptide	0.389	0.330	down
Tyr-Glu	Small Peptide	0.343	0.456	down

Compounds	Class	Fold change (LFD+EtOH/LFD)	Fold change (HFD+EtOH/HFD)	Changes*
Phe-Trp	Small Peptide	0.185	0.567	down
Met-Met	Small Peptide	0.159	0.629	down
His-Ala	Small Peptide	0.256	0.703	down
Tryptamine	Indole and Its derivatives	0.313	0.128	down
β-indole-3-acetic acid	Indole and Its derivatives	0.679	0.401	down
Nonanoic Acid	Fatty Acyls	0.691	0.549	down
Succinic Acid	Organic acid And Its derivative Tetradecanedioic acids	0.311	0.552	down
Ferulic Acid	Organic acid And Its derivatives	0.705	0.535	down
5-Aminovaleric Acid	Organic acid And Its derivatives	0.531	0.596	down
2-Hydroxyglutaric acid	Organic acid And Its derivatives	0.290	0.420	down
N-formylkynurenine	Organic acid And Its derivatives	0.597	0.685	down
Tetradecanedioic acid	Fatty Acyls	0.506	0.310	down

*, Fold change (FC) threshold of less than 0.75 or greater than 1.5 as the criterion for up or down.

Table S2. Primers for the quantification of inflammatory cytokines, lipid metabolism-related genes, and liver fibrosis-related genes in mice.

Gene	Forward （5'-3'）	Reverse （5'-3'）
GAPDH	CTCTGGAAAGCTGTGGCGTGATG	ATGCCAGTGAGCTTCCCGTTCAG
Tnfa	CCAAAGGGATGAGAAGTTCC	CTCCACTTGGTGGTTTGCTA
Ccl2	TGCTCGTGGCTGCCTTCTGT	TGTGAAGCTGCCGGGAGGTGTA
Fabp1	AGGAGTGCGAACTGGAGACCAT	GTCTCCATTGAGTTCAGTCACGG
Cd36	GGACATTGAGATTCTTTTCCTCTG	GCAAAGGCATTGGCTGGAAGAAC
Pparγ	GTACTGTCGGTTTCAGAAGTGCC	ATCTCCGCCAACAGCTTCTCCT
Srebp1c	CGACTACATCCGCTTCTTGCAG	CCTCCATAGACACATCTGTGCC
Acc	GTTCTGTTGGACAACGCCTTCAC	GGAGTCACAGAAGCAGCCCATT
Fas	AGCACTGCCTTCGGTTCAGTC	AAGAGCTGTGGAGGCCACTTG
Pparα	ACCACTACGGAGTTCACGCATG	GAATCTTGCAGCTCCGATCACAC
Cpt1a	GGCATAAACGCAGAGCATTCCTG	CAGTGTCCATCCTCTGAGTAGC
Acta2	GAGCATCCGACACTGCTGAC	GCACAGCCTGAATAGCCACA
Timp1	AGGTGGTCTCGTTGATTTCT	GTAAGGCCTGTAGCTGTGCC
Col3a1	GACCAAAAGGTGATGCTGGACAG	CAAGACCTCGTGCTCCAGTTAG

Table S3. Comparison of main outcomes between alcohol-treated lean and obese mice.

Category	Metric	Lean Mice (LFD+EtOH)	Obese Mice (HFD+EtOH)
Basic Phenotypes	Blood ethanol	↑	↑
	Body weight	↓*NS	↓*NS
	Food intake	↓	↓
	Liver weight	↑*NS	↑
	Liver index	↑	↑
	Serum TG	↑	↑*NS
	Serum ALT	*NS	↓
	Fasting glucose	↓	*NS
Hepatic Steatohepatitis	Hepatic TG	↑	*NS
	Hepatic CHO	↑	*NS
	Cd36，Acc，Fas	↑	↑
	Srebp1c	*NS	↑
Inflammation and Fibrosis	Neutrophil marker: CD11B	↑	↑
	Macrophage marker: F4/80	*NS	↑
	Pro-inflammatory gene: Tnfα, Ccl2	↑*NS	↑
	Pro-fibrotic gene: Col3a1, Acta2, Timp1	*NS	↑
	Fibrotic area	↑*NS	↑
Gut Microbiota and Metabolites	ASV	↑	↑
	α-diversity	↑	↑
	SCFAs-producing bacterial: Dubosiella	↓	↓ *NS
	SCFAs-producing bacterial: Ileibacterium	↓ *NS	↓
	Atherosclerosis-promoting bacterial: Lachnoclostridium	*NS	↑
	Lipid-lowering SCFAs: Caproic acid	↓	↓ *NS
	Lipid-lowering SCFAs: Ferulic acid	↓ *NS	↓
	Anti-inflammatory amino acids:	Glutamine↓, Histidine↓*NS, Glycine↓	Glutamine↓*NS, Histidine↓, Glycine↓*NS
	KEGG predictive changes	Amino acid metabolism pathways	Cholesterol metabolism and inflammatory pathways

↑: Increased content; ↓: Decreased content; *NS: No significant difference; ↑*NS: Increased level with no significant difference; ↓*NS: Decreased level with no significant difference.
